# Identification of two novel *SALL1* mutations in chinese families with townes-brocks syndrome and literature review

**DOI:** 10.1186/s13023-023-02874-4

**Published:** 2023-08-29

**Authors:** Zhendong Wang, Zhenfu Sun, Yujie Diao, Zhouyang Wang, Xiangdong Yang, Bei Jiang, Yumei Wu, Guangyi Liu

**Affiliations:** 1 Department of Nephrology, Qilu Hospital, Shandong University, Jinan, China; 2 Department of Nephrology, Jining NO.1 People’s Hospital, Jining, China; 3 Department of Nephrology, Heze Municipal Hospital, Heze, China

**Keywords:** Townes-Brocks syndrome, Whole-exome sequencing, *SALL1*, Alphafold, Genotype, Renal phenotype

## Abstract

**Background:**

Townes-Brocks syndrome is a rare autosomal dominant genetic syndrome caused by mutations in *SALL1*. The clinical features of Townes-Brocks syndrome are highly heterogonous. Identification of new *SALL1* mutations and study of the relation between *SALL1* mutations and clinical features can facilitate diagnosis of Townes-Brocks syndrome.

**Methods:**

We collected clinical data and blood samples of the two patients and their family members for whole-exome sequencing and Sanger sequencing. Prediction analysis of the SALL1variation protein structure was achieved using Alphafold. The clinical materials and gene sequencing results were analyzed. The clinical materials and gene sequencing results were analyzed. The related literature of Townes-Brocks syndrome were searched and the genotype-renal phenotype analysis was performed combined with this two cases.

**Results:**

Based on the clinical features and gene sequencing results, the two patients were diagnosed as Townes-Brocks syndrome. Two novel *SALL1* mutations (c.878-887del and c.1240G > T) were identified, both of which were pathogenic mutations. The correlation between genotypes and renal phenotypes in Townes-Brocks syndrome patients caused by *SALL1* mutation were summarized.

**Conclusion:**

This study identified two novel mutations and provided new insights into the correlation of genotypes and renal phenotypes of Townes-Brocks syndrome.

**Supplementary Information:**

The online version contains supplementary material available at 10.1186/s13023-023-02874-4.

## Introduction

Townes-Brocks syndrome (TBS; OMIM 104,780) was first described by Townes and Brocks in 1972 as a rare autosomal dominant malformation syndrome [1]. Till now, more than 100 cases of TBS have been reported. The incidence of TBS is 1:250,000 [2]. The clinical features of TBS patients are highly heterogeneous, with the main clinical features being anal atresia, external ear dysplasia and thumb deformity [2]. Other clinical features may include congenital heart defects, malformations of the renal or genitourinary system, eye abnormalities, endocrine abnormalities and growth retardation [3]. TBS is caused by mutations in the zinc finger transcription factor *SALL1* [4]. SALL1 protein is one of the four members of the evolutionarily conserved SALL protein family that are essential for organogenesis. SALL1 protein is mainly expressed in brain, liver and kidney and highly expressed during embryonic development [5–8]. It is an important regulator in the development of the urinary system, limbs, ears, brain and liver. *SALL1* mutations can cause malformation of the anus, outer ear, limbs and urogenital system by altering the protein spatial structure [9]. Different types of *SALL1* mutations have been reported in TBS patients, including nonsense mutation, frameshift mutation, gene deletion, duplication and insertion [7]. The clinical features and severity of TBS caused by different mutations are often different. Here, we reported two patients with TBS, one with renal failure, polycystic nephropathy, external ear dysplasia, and the other one with renal failure and external ear dysplasia. The clinical and genetic characteristics of the two cases are summarized. Based on these two cases and previously reported TBS cases, we analyzed the relation between *SALL1* mutations and renal phenotypes in TBS patients.

## Materials and methods

### Clinical investigations

The medical history and physical examination results of patients and their family members were collected. The pedigree of family was drawn. The patients were given blood routine, urine routine, liver and renal function, thyroid function and limb X-ray examination.

### DNA sample collection

This study was approved by the Ethics Committee of Qilu Hospital of Shandong University and conducted in accordance with the Declaration of Helsinki. Informed consent was obtained from all the patients and their families.

DNA was extracted from fasting blood samples using a genomic DNA kit according to the manufacturer’s instruction (Vazyme Fast Pure Blood DNA Isolation Mini Kit V2).

### Whole-exome sequencing

The patients’ DNA samples were sent to Fujun Gene Sequencing for whole-exome sequencing. The library was constructed using KAPA Library Preparation Kit. The target sequences were captured and enriched using the on-chip exon capture system. The high-throughput sequencing was performed on Illumina NovaSeq. The sequencing data were evaluated and qualified by Illumina Sequence Control Software (SCS).

### Bioinformatic analysis

Raw sequencing data were processed and analyzed by Illumina Pipeline version (version 1.3.4) software, filtered to remove contamination and compared with the BWA software package for clean reads. Insertion and deletion mutation were identified by GATK Indel Genotyper, and SNPS were identified by SOAP snp software. The control population databases used in the data flow analysis included the 1000 Genomes database, dbSNP database and locus specific databases. The effect of gene mutation on protein spatial structure was predicted. The conservation of mutation sites was calculated. The identified mutations were searched in the Human Gene Mutation Database (HMGD) and Clinver database. The pathogenicity of mutation was assessed using the American College of Medical Genetics and Genomics (ACMG) guidelines [9].

### Sanger sequencing

The regions where the mutations resided in were amplified by PCR. The primers were designed using Premier 5 software. The PCR products were applied to Sanger sequencing.

### Literature review

The PubMed database was searched with “*SALL1*” and “Townes Brocks syndrome” as keywords. All literatures are published in English and freely available. All cases of Townes-Brocks syndrome with *SALL1* mutation were reviewed.

## Result

### Clinical features of the patients

#### Patient 1

**Fig. 1:**
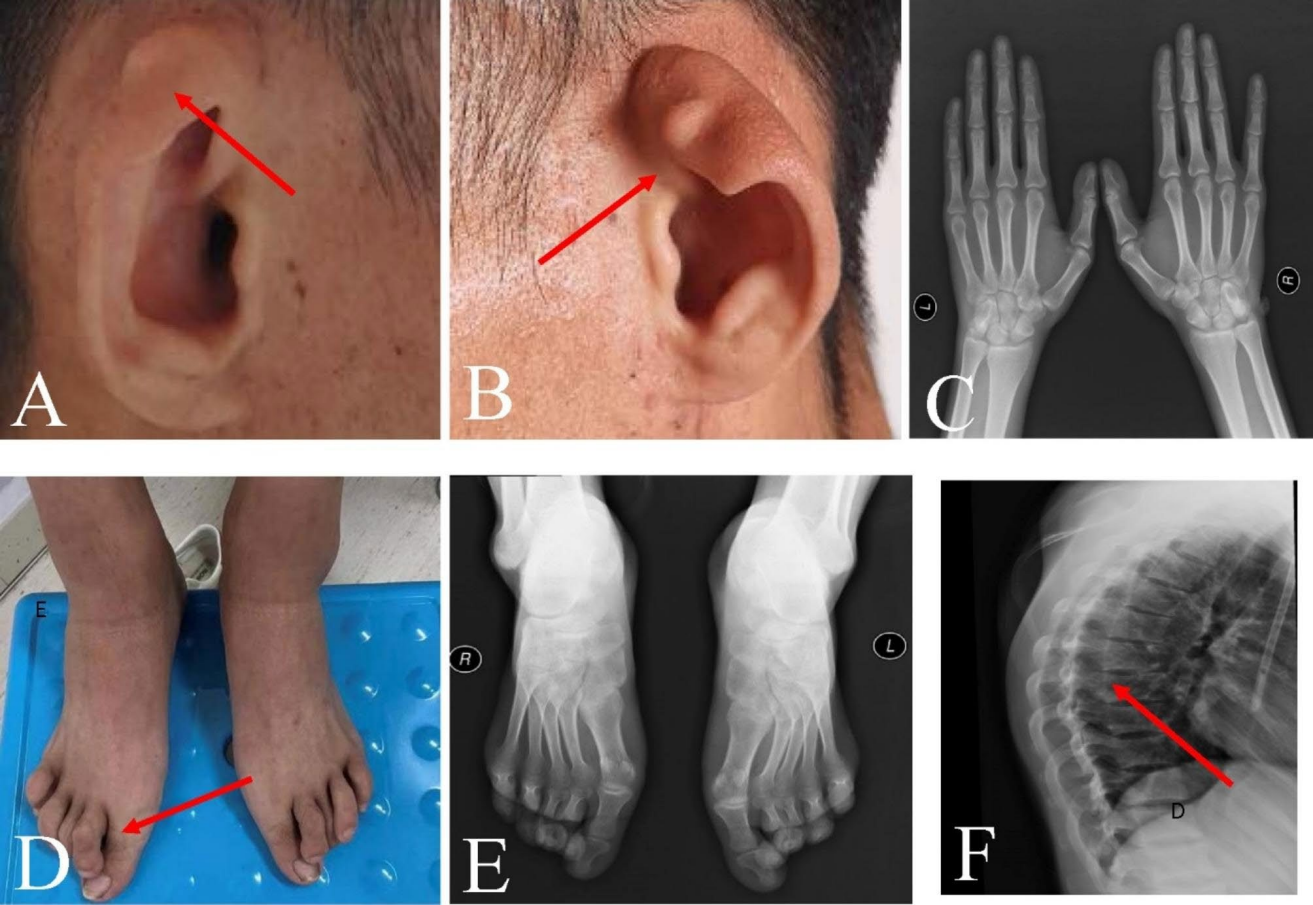
Clinical Features of Patient 1 with Townes-Brocks Syndrome **A**: Outer ear tag of the right ear (red arrow), **B**: Outer ear tag of the left ear (red arrow), **C**: X-ray of both hands, **D**: toe overlap (red arrow), **E**: X-ray of both feet, **F**: Posterior process of thoracic vertebra (red arrow)

**Fig. 2:**
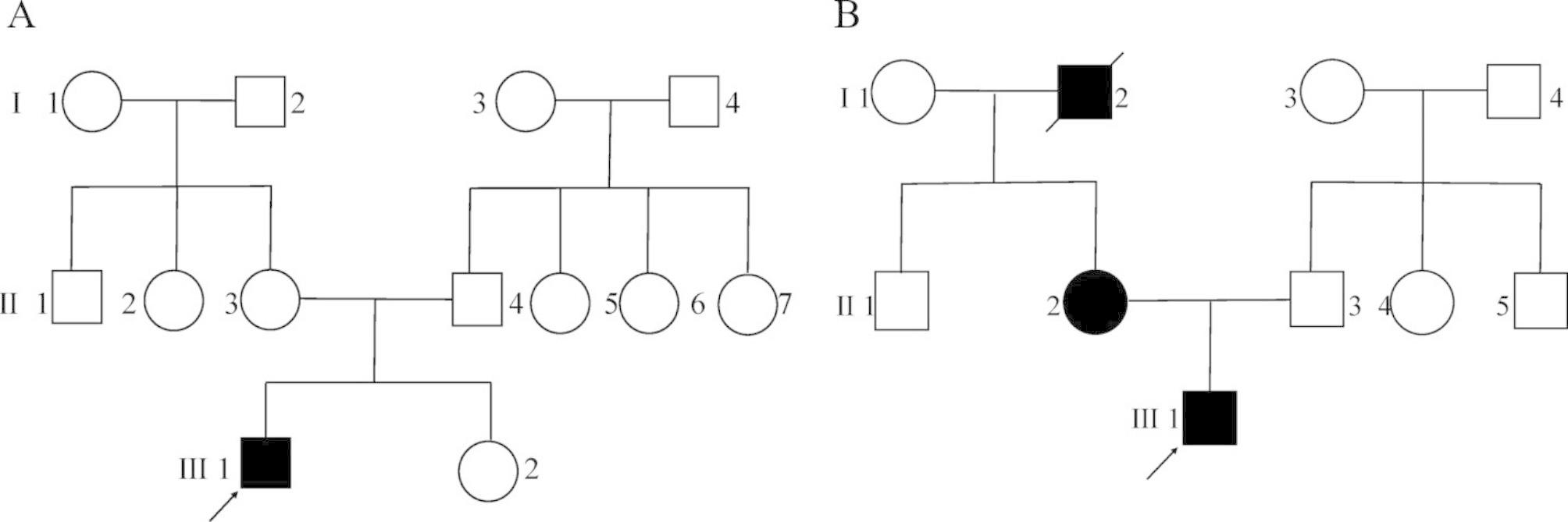
Pedigree family of TBS patients **A**: Pedigree family of Patient 1; **B**: Pedigree family of Patient 2

The proband (III-1) was a 27-year-old male with chronic renal insufficiency for more than 9 years. The patient’s renal ultrasound examination performed 9 years ago revealed reduced volume in both kidneys. Blood test and urine test conducted one year ago showed that the level of serum creatinine was 459 µmol/L, hemoglobin was 119 g/L and urine protein was ++. The patient had a history of hypertension for more than one year. The patient received routinely hemodialysis and antihypertensive treatment. Physical examination revealed external ear tags (Fig. 1A, B), overlapping toes (Fig. 1D), normal X-rays of hands and feet (Fig. 1C, E), posterior thoracic process (Fig. 1F). Renal ultrasound showed reduced renal volume with multiple cysts in both kidneys. Pure tone electroaudiometry suggested conductive hearing loss. No obvious abnormalities were identified in fundus and cardiac ultrasound examination. According to the patient’s past medical history and examination, no primary or other secondary renal diseases were found. According to the composition of the patient’s family, the family pedigree was drawn (Fig. 2A). There were 13 members in three generations of this family. All the other family members were normal. The parents were not consanguineous.

#### Patient 2

The proband (III-1) presented with “chronic renal insufficiency” was a 14-year-old male. This patient had bilateral external ear dysplasia and secondary hyperparathyroidism. The level of serum creatinine was 600 µmol/L. There were no obvious abnormalities in hands and feet. His hearing was normal. The patient’s mother and maternal grandfather also had ear malformation and proteinuria. The patient’s father did not have any of the abovementioned clinical features. The family pedigree was drawn (Fig. 2B). There were 10 members in three generations of this family. Except for the proband (III-1), the patient’s mother (II-3) and maternal grandfather (I-2), all the other family members were normal. The parents were not consanguineous.

### Whole-exome sequencing and Sanger sequencing

**Fig. 3:**
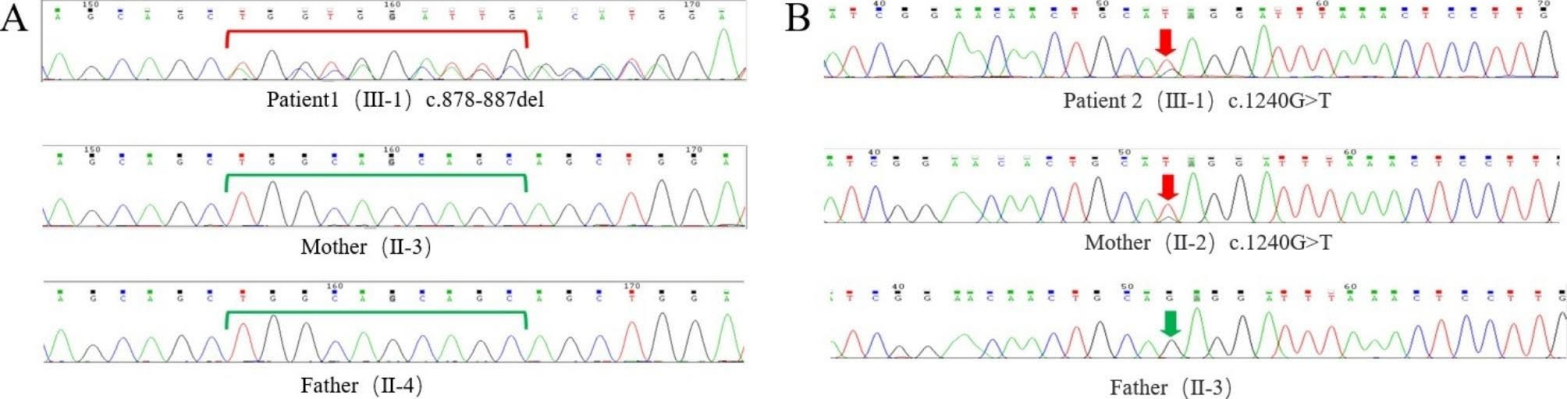
Sanger sequencing of patients and family members **A**: Sanger sequencing of patient 1 and family members; **B**: Sanger sequencing of patient 2 and family members

Blood samples were collected from the two patients. Whole-exome sequencing identified a frameshift mutation at *SALL1* (c. 878-887del) in patient 1, which was not detected from his parents by Sanger sequencing (Fig. 3A). Patient 2 had a nonsense mutation c.1240G > T in *SALL1* gene. Sanger sequencing indicated that the mutation was inherited from the patient’s mother (Fig. 3B).

### Bioinformatic analysis

**Fig. 4:**
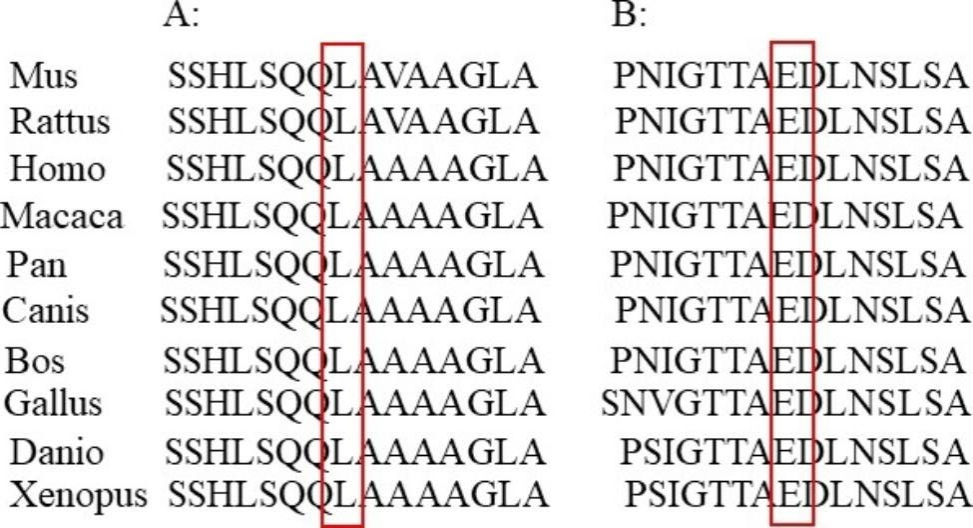
SALL1 variation protein are conserved in different vertebrates **A**: Leucine 293 is conserved in different vertebrates; **B**: Glutamic 414 is conserved in different vertebrates

**Fig. 5:**
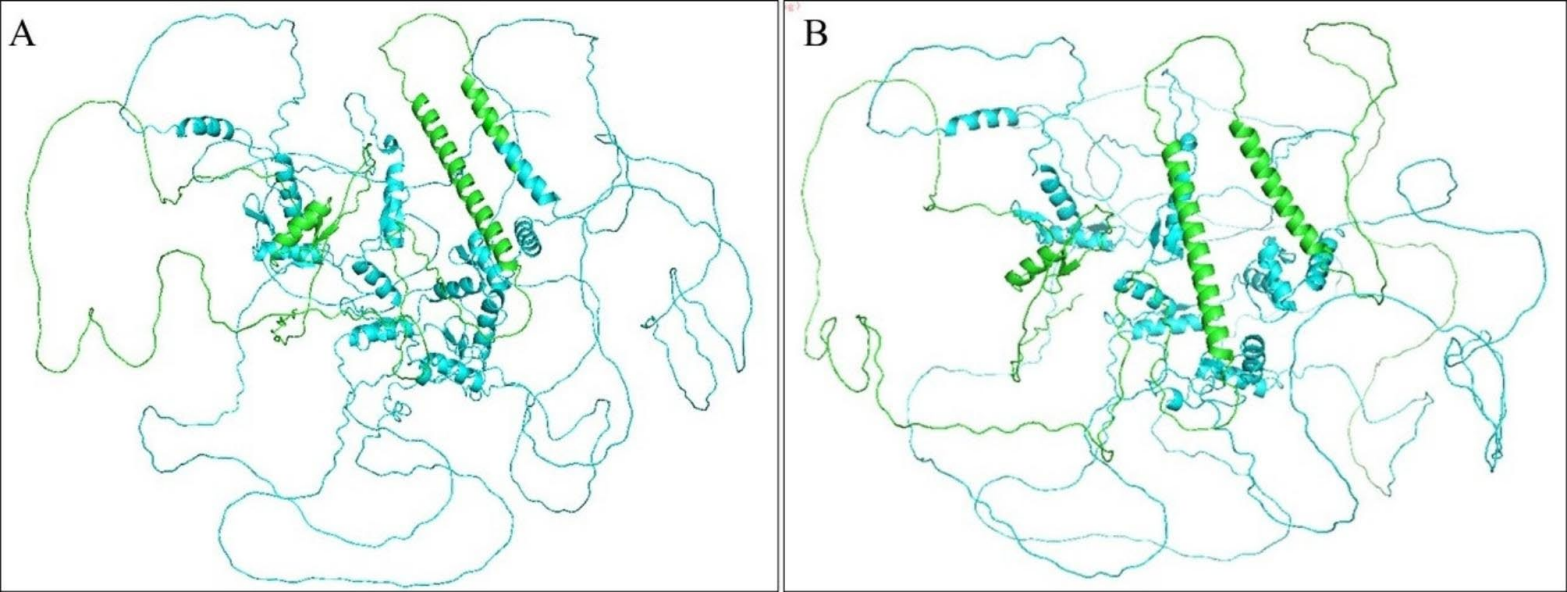
Prediction analysis of SALL1 protein structure **A**: The SALL1 (p.Leu293Glnfs*18 protein structure ; **B**: The SALL1 (p.E414X) protein structure Protein consequences of *SALL1* mutation. Prediction analysis of the SALL1 (p.Leu293Glnfs*18 and p.E414X) protein structure was achieved using Alphafold and visualized using PyMOL. Prediction analysis of the SALL1 (p.Leu293Glnfs*18 and p.E414X) protein structure shown the amino acid sequence structure changed significantly and some domains were lost. The green part represents the common amino acid sequence of normal. The blue part represents the amino acid sequence changes and protein truncation

The c.878-887del mutation in *SALL1* of patient 1 resulted in mutation of leucine 293 to glutamine and deletion of 3 amino acids afterward in SALL1 protein. The c.1240G > T mutation identified in patient 2 resulted in an early termination of SALL1 translation after the first 413 amino acids. *SALL1* mutations(c.878-887del and c.1240G > T)were not reported in HMGD and Clinver databases. Leucine 293 and Lysine are conserved in SALL1 protein across different vertebrates (Fig. 4). Structure prediction analysis of SALL1 protein showed that the c.878-887del and c.1240G > T mutations caused loss of some domains (Fig. 5). According to ACMG, the *SALL1* (c. 878-887del) allele was rated as P = PVS1 + PS2 + PM2 and the *SALL1* (c.1240G > T) allele was rated as P = PVS1 + PM1 + PM2. The two novel *SALL1* mutations were pathogenic.

### Literature review on the relation between SALL1 mutations and renal phenotypes in TBS patients

**Fig. 6:**
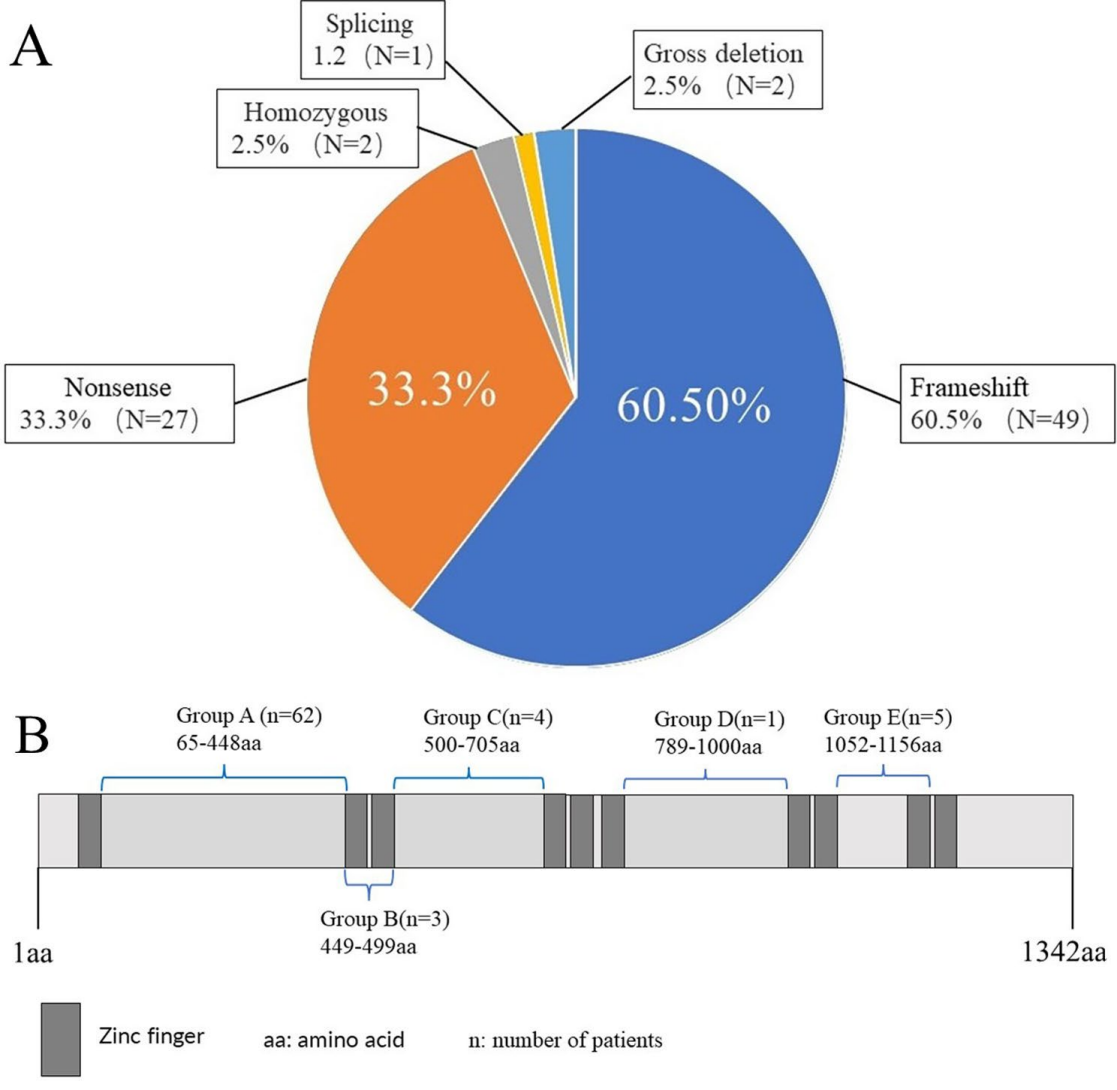
Mutation types and region of SALL1 protein variation in different groups **A**: Summary of mutation types in TBS patients with kidney disease; **B**: The region of SALL1 protein variation in different groups

The literature of TBS patients with renal abnormalities reported from May 1984 to March 2022 were reviewed. And the *SALL1* mutations and renal phenotypes were summarized (Fig. 6, Supplementary Table S1). We reviewed eighty-one affected individuals from fifty-two families, including forty-one males (50.6%), thirty-seven females (45.6%) and three (3.8%) with no gender reported. The ratio of male to female was 1.11:1. Forty-eight *SALL1* mutations were reported in these eighty-one patients, including forty-nine frameshift mutations (60.5%), twenty-seven nonsense mutations (33.3%), one splicing mutation (1.2%), two gross deletions (deletion of more than 20 bp) (2.5%), and two homozygous mutation (2.5%) (Fig. 6A). 64% of them had renal structural abnormalities [10]. 28% of the patients had serum creatinine levels above the normal range but did not meet the diagnosis of renal failure, hence, were considered having renal injury. Five patients underwent successful kidney transplants and two of them developed graft rejection [11–14]. The patients were grouped based on the location of the *SALL1* mutations they carried (Fig. 6B). The *SALL1* mutations and renal features of the TBS patients with kidney disease were summarized in Table 1. Phenotypic classification was based on the zinc finger domains [13, 15]. There were sixty-two patients in group A, accounted for 76.54% of all reported TBS patients with kidney disease. In group A, the average age of the TBS patients with renal failure was 23 years old and the median age was 19 years old. However, there were no cases with abnormal renal function in groups C and D. In group E, there were three patients with renal failure. One had renal failure after the age of 32, and the other two had renal failure at the age of 52. Group F contained one patient with severe renal failure caused by a splice mutation in *SALL1* (c. IVS2-19T > A). Group G consisted of two families with gross deletions and all patients developed renal failure [16, 17]. Another case of *SALL1* (c.3160 C > T) was autosomal recessive inheritance and the patient started dialysis treatment when she was 7 months old in Group H [18].

**Table 1:** Details of mutation types and renal phenotypes in different groups of TBS patients with kidney disease

Group	Mutation	RAV	Total	Gender			RF	RI	HPL	PK	VR	HSP
				Male	Female	Unspecified						
**A**	Frameshift	65–448	38	21	16	1	5	14	28	4	6	4
	Nonsense		24	11	11	2	5	9	12	3	4	5
**B**	Frameshift	449–499	4	3	1	0	1	1	3	1	1	0
	Nonsense		0	0	0	0	0	0	0	0	0	0
**C**	Frameshift	500–705	2	0	2	0	0	0	2	0	2	0
	Nonsense		2	0	2	0	1	0	2	0	0	0
**D**	Frameshift	789–1000	1	0	1	0	0	0	0	0	0	0
	Nonsense		0	0	0	0	0	0	1	0	0	1
**E**	Frameshift	1052–1156	5	4	1	0	3	0	3	1	1	0
	Nonsense		0	0	0	0	0	0	0	0	0	0
**F**	Splicing	N/A	1	1	0	0	1	0	1	0	0	0
**G**	Gross deletion	N/A	2	1	1	0	2	0	0	0	1	0
**H**	Homozygous	N/A	2	0	2	0	1	0	1	2	0	0

RAV, The range of amino acids in which the variation occurs; N/A, Not applicable. RF, Renal failure (Patients had received renal replacement therapy or creatinine > 707µmol/L or eGFR ≤ 15ml/min/1.73m^2^.); RI, Renal injury (the patient’s serum creatinine levels were above the normal range but didn’t meet the diagnosis of renal failure); HPL, hypoplastic; PK, Polycystic kidney; VR, Vesicoureteral reflux; HSP, hypospadias. Gross deletions are currently defined as > 20 bp, which is a crude cut-off value

## Discussion

TBS is an autosomal dominant malformation syndrome caused by *SALL1* mutations and approximately one in three patients have not family history with Townes-Brocks syndrome. *SALL1* containing three exons and two introns locates on chromosome 16q12.1 and encodes SALL1 protein [19]. SALL1 protein is a zinc finger transcription factor containing four highly conserved C2H2 double zinc finger domains and an alanine and glutamine rich domain [20]. *SALL1* mutations lead to TBS mainly through dominant negative effect and haploinsufficiency. Most *SALL1* mutations are located in exon 2 or intron 2, especially in the 5’ end or within the first double zinc finger coding region, resulting in truncated SALL1 protein lack the lack the zinc-finger domain that mediates chromatin-DNA interactions, but retain the N-terminal domain [19]. These truncated proteins can affect the development of heart and limb by dominant negative effect [21, 22] .Mice expressing SALL1 truncated protein are more prone to TBS phenotype, possibly due to the truncated protein interacts with other SALL family member proteins and interfere their functions [23]. Recent studies have also shown that TBS may be a ciliary disease. The truncated SALL1 proteins can affect the normal function of primary ciliary regulatory proteins CCP110 and CEP97 and increase LUZP1 degradation. This not only leads to abnormal primary cilia morphology and growth frequency in cells, but also destroys the formation and function of cilia. Thus, some patients with *SALL1* mutations have symptoms similar to ciliopathies such as polycystic kidney disease and hearing loss occur [23]. The first patient of this study had multiple cysts in kidneys.

HGMD has collected 116 *SALL1* mutations, including frameshift mutations, nonsense mutations, gross deletions and splice mutations until March 2022. Most of the mutations reported in TBS patients are frameshift mutations or nonsense mutations located in the mutational hotspot region, which is between nucleotide 764 and nucleotide 1565. This 802 bp region encodes the glutamine-rich interaction domain and the most amino terminal double zinc finger domain [23]. Mutations in the hotspot region mainly result in truncated SALL1 protein [23–25]. The frameshift mutations or nonsense mutations located in the mutation hotspot region lead to classical or more severe TBS phenotypes, while the clinical features caused by insufficient haploid expression of *SALL1* is relatively mild [7]. The two novel mutations (c.878-887del and c.1240G > T) identified in this study were located in the hotspot mutation region of *SALL1*. Both Patient 1 and Patient 2 had external ear malformation and renal function impaired, but the renal dysfunction in patient 2 was more severe than that in patient 1.

Patient 1 carried a novel frameshift mutation (c.878-887del) in the hotspot mutation region of *SALL1.* This mutation resulted in a frameshift at amino acid 293 and a truncated SALL1 protein (p.Leu293Glnfs*18). This mutation is a de novo mutation. All the other members in Patient1’s family were normal. Patient 2 showed mild classic clinical symptoms of TBS. Only external ear dysplasia was observed and no dysplasia such as anal atresia and finger deformity. The patients with *SALL1* mutations (c.419delC) have similar symptoms such as external ear dysplasia, finger deformity and renal function impaired [15]. Whole-exome sequencing identified a novel nonsense mutation (c.1240G > T) in the hotspot mutation region of *SALL1*, resulting in premature termination after amino acid 414.

SALL1 protein is highly expressed in the embryonic kidneys and participates in renal development by regulating the expression of major renal development genes (*PAX8*, *GDNF* and *FOXD1*) [26]. *SALL1* mutations affect kidney structure and function through dominant negative effects and haploinsufficiency. About 40% of TBS patients have some renal abnormalities, such as renal dysplasia, polycystic kidney, peripheral bladder reflux and hypospadias [26]. Different members in the same family may have different clinical features. In the most families, the clinical features of the offspring are more serious. The underlying mechanism is not clear [27, 28]. SALL1 protein has two key transcriptional repressor domains, consisting of the N-terminal 1–87 amino acids and 434–690 amino acids, respectively. Loss of either transcriptional repressor domain or disruption of the integrity of region 434–690 amino acids significantly reduces the transcription repression activity of SALL1 protein. The region after amino acid 690 had little effect on SALL1 activity [27]. These may explain why renal failure in patients of group A occurred much earlier than that in group F, and the patients in groups C and D had no abnormal renal function. Therefore, the location of *SALL1* mutations is correlated with the severity of the renal phenotype. The patient in group F carried c.IVS2-19T > A mutation in intron 2, leading to abnormal splicing and premature termination of SALL1 protein (1208 aa) [29], indicating that the pathogenic *SALL1* mutations exist not only in exons, but also in introns. Group G consisted of two families with renal failure. One of them had partial fragment deletion of the single allele of *SALL1* (del3384bp), which resulted in deletion of a key transcriptional repressor domain of SALL1 protein [17]. The other had partial fragment deletion of *SALL1* (del6Mb), which resulted deletion of other genes related to kidney disease [16]. The heterozygous *SALL1* mutation (c.3160 C > T) can produce a small amount of truncated protein, which may retain part of SALL1 protein function. Therefore, the carriers of *SALL1* (c.3160 C > T) heterozygous mutation do not show obvious phenotypes. It is generally suggestion that TBS is autosomal dominant disease. However, the homozygous *SALL1* mutation (c.3160 C > T) in 2 female siblings with renal failure, multiple congenital anomalies, central nervous system defects, and cortical blindness was reported [18] .

Based on the above analysis, we made the following conclusions. (1) Different members can have different clinical features in the same family. The clinical features are usually more severe in the offspring of the same family. (2) TBS patients with *SALL1* mutations in the coding region for the key transcriptional repressor domain have more severe renal phenotypes. (3) Mutations affecting the 500–1156 amino acids of SALL1 protein are less likely to cause renal phenotypes in TBS patients. (4) Patients with *SALL1* splicing mutations or gross deletions are prone to develop severe renal failure. (5) TBS has autosomal recessive inheritance. Homozygous mutants are more likely to have severe renal phenotypes than heterozygous mutation.

## Conclusion

In this study, two novel *SALL1* (c.878-887del and c.1240G > T) were identified in two TBS patients. These two mutations are both located in the hotspot mutations region of *SALL1*, which is consistent with the severe renal phenotypes. Most *SALL1* mutations locate in the hotspot mutations region, and renal failure is more likely to occur in patients with these *SALL1* mutations. However, more TBS patients are needed to understand the correlation between genotypes and renal phenotypes.

### Electronic supplementary material

Below is the link to the electronic supplementary material.

Supplementary Material 1

## Data Availability

All data generated or analyzed during this study are included in the article, further inquiries can be directed to the corresponding author. The two novel variants have been. submitted to the Clinvar (https://www.ncbi.nlm.nih.gov/clinvar/).

## Abbreviations

**TBS**: Townes-Brocks syndrome
**ACMG**: American College of Medical Genetics and Genomics
**HMGD**: Human Gene Mutation Database
**SCS**: Illumina Sequence Control Software
**SALL1**: Sal-like protein 1
