# Supplementary material for: Identification of two novel SALL1 mutations in chinese families with townes-brocks syndrome and literature review
Source: Orphanet J Rare Dis. 2023 Aug 29;18:250. doi: 10.1186/s13023-023-02874-4 (PMC10466882; doi:10.1186/s13023-023-02874-4)
Supplement: Supplementary file 1 — Supplementary Material 1 [file 13023_2023_2874_MOESM1_ESM.docx]

*Supplementary Material*

Identification of Two Novel *SALL1* Mutations in Chinese Families with Townes-Brocks syndrome and Review of the Literature

Zhendong Wang, Zhenfu Sun, Yujie Diao, Zhouyang Wang, Xiangdong Yang, Bei Jiang, Yumei Wu, Guangyi Liu ^*^

***Correspondence:** Guangyi Liu [guangyi.liu@sdu.edu.cn](mailto:guangyi.liu@sdu.edu.cn)

# Supplementary Tables

**Table -1 Genotypes and Renal phenotypes in 81 Townes-Brocks syndrome patients**

| **Genetic finding** | **N** | | **Gender** | **Phenotype** | | | | | | **Ref** |
| --- | --- | --- | --- | --- | --- | --- | --- | --- | --- | --- |
|  |  |  |  | **RF** | **RFI** | **HPL** | **PK** | **VR** | **HSP** |  |
| [**Frameshift**](javascript:;) |  | |  |  |  |  |  |  |  |  |
| c.313delA | 1 | | F | 0/1 | 1/1 | 0/1 | 0/1 | 0/1 | 0/1 | [1] |
| c.419delC | 1 | | M | 0/1 | 1/1 | 1/1 | 0/1 | 0/1 | 0/1 | [2] |
| c.764delT | 1 | | M | 0/1 | 1/1 | 0/1 | 1/1 | 0/1 | 0/1 | [1] |
| c.792delGC | 2 | | F/M | 0/2 | 0/2 | 1/2 | 0/2 | 2/2 | 0/2 | [3] |
| c.817delG | 1 | | M | 1/1 | 0/1 | 1/1 | 0/1 | 0/1 | 0/1 | [4] |
| c.899del2 | 2 | | F/M | 0/2 | 0/2 | 2/2 | 0/2 | 1/2 | 0/2 | [3] |
| c.981_982insTGGC | 1 | | M | 0/1 | 1/1 | 1/1 | 1/1 | 0/1 | 0/1 | [5] |
| c.995delC | 1 | | M | 0/1 | 0/1 | 1/1 | 0/1 | 0/1 | 0/1 | [1] |
| c.1028_1029delTA | 1 | | M | 0/1 | 0/1 | 0/1 | 0/1 | 0/1 | 1/1 | [6] |
| c.1047dupC | 1 | | M | 0/1 | 0/1 | 0/1 | 0/1 | 1/1 | 1/1 | [7] |
| c.1119_1197del79 | 1 | | F | 1/1 | 0/1 | 1/1 | 0/1 | 0/1 | 0/1 | [1] |
| c.1134delT | 1 | | F | 0/1 | 1/1 | 1/1 | 0/1 | 0/1 | 0/1 | [1] |
| c.1146delT | 3 | | 2F/M | 0/3 | 3/3 | 2/3 | 0/3 | 0/3 | 0/3 | [2] |
| c.1145_1146insTA | 1 | | M | 0/1 | 1/1 | 0/1 | 1/1 | 0/1 | 1/1 | [6] |
| c.1174_1175del2 | 1 | | M | 0/1 | 0/1 | 1/1 | 0/1 | 0/1 | 0/1 | [1] |
| c.1200_1206del7 | 1 | | M | 1/1 | 0/1 | 1/1 | 0/1 | 0/1 | 0/1 | [2] |
| c.1263delC | 1 | | F | 0/1 | 1/1 | 1/1 | 0/1 | 0/1 | 0/1 | [6] |
| c.1277del2 | 1 | | F | 0/1 | 1/1 | 0/1 | 0/1 | 0/1 | 0/1 | [2] |
| c.1291del10 | 2 | | 2M | 0/2 | 2/2 | 2/2 | 0/2 | 0/2 | 0/2 | [2] |
| c.1321dupA | 1 | | F | 0/1 | 1/1 | 1/1 | 1/1 | 0/1 | 0/1 | [6] |
| c.1326delC | 2 | | 2F | 0/2 | 0/2 | 2/2 | 0/2 | 0/2 | 0/2 | [6] |
| c.1327delG | 2 | | M/？ | 0/2 | 0/2 | 2/2 | 0/2 | 0/2 | 0/2 | [6] |
| c.1347delCA | 1 | | M | 0/1 | 0/1 | 1/1 | 0/1 | 0/1 | 0/1 | [8] |
| c.1404dupG | 9 | | 4F/5M | 2/9 | 0/9 | 6/9 | 0/9 | 2/9 | 1/9 | [6] |
| c.1415_1425del11 | 1 | | F | 0/1 | 0/1 | 1/1 | 0/1 | 0/1 | 0/1 | [1] |
| c.1451_1458del7insT | 1 | | M | 0/1 | 1/1 | 1/1 | 1/1 | 0/1 | 0/1 | [5] |
| c.1470delG | 1 | | M | 1/1 | 0/1 | 0/1 | 0/1 | 1/1 | 0/1 | [9] |
| c.1516_1517dupAT | 2 | | 2F | 0/2 | 0/2 | 2/2 | 0/2 | 2/2 | 0/2 | [1] |
| c.3437delG | 3 | | 1F/2M | 3/3 | 0/3 | 1/3 | 1/3 | 0/3 | 0/3 | [10] |
| c.3249_3255del7 | 1 | | M | 0/1 | 0/1 | 1/1 | 0/1 | 1/1 | 1/1 | [1] |
| c.3414delAT | 1 | | M | 0/1 | 0/1 | 1/1 | 0/1 | 0/1 | 0/1 | [1] |
| **Total** | 49 | | 20F/28M/? | 9/49 | 14/49 | 35/49 | 6/49 | 10/49 | 5/49 |  |
| **Nonsense** |  | |  |  |  |  |  |  |  |  |
| c.727C > T | 2 | | 2F | 2/2 | 0/2 | 0/2 | 0/2 | 0/2 | 0/2 | [11] |
| c.814C>T | 2 | | F/M | 0/2 | 1/2 | 2/2 | 0/2 | 0/2 | 0/2 | [1] |
| c.824T>G | 1 | | M | 0/1 | 1/1 | 0/1 | 0/1 | 1/1 | 0/1 | [12] |
| c.826C>T | 9 | | 4F/3M/2? | 1/9 | 3/9 | 5/9 | 2/9 | 0/9 | 2/9 | [2, 8, 13-15] |
| c.874C>T | 1 | | M | 1/1 | 0/1 | 1/1 | 1/1 | 0/1 | 0/1 | [16] |
| c.967C>T | 3 | | 3M | 0/3 | 2/3 | 1/3 | 0/3 | 1/3 | 3/3 | [17] |
| c.1112C>G | 1 | | M | 0/1 | 1/1 | 0/1 | 0/1 | 1/1 | 0/1 | [18] |
| c.1115C>G | 2 | | F/M | 0/2 | 0/2 | 1/2 | 0/2 | 1/2 | 0/2 | [4] |
| c.1228G>T | 1 | | F | 1/1 | 0/1 | 0/1 | 0/1 | 0/1 | 0/1 | [1] |
| c.1256T>A | 2 | | 2F | 0/2 | 1/2 | 2/2 | 0/2 | 0/2 | 0/2 | [1] |
| c.1509C>A | 1 | | F | 0/1 | 0/1 | 1/1 | 0/1 | 0/1 | 0/1 | [19] |
| c.1519C > T | 1 | | F | 1/1 | 0/1 | 1/1 | 0/1 | 0/1 | 0/1 | [20] |
| c.2779C>T | 1 | | F | 0/1 | 0/1 | 1/1 | 0/1 | 0/1 | 0/1 | [21] |
| **Total** | 27 | 14F/11M/2？ | | 6/27 | 9/27 | 15/27 | 3/27 | 4/27 | 5/27 |  |
| **Splicing** |  | |  |  |  |  |  |  |  |  |
| IVS2/19T | 1 | | M | 1/1 | 0/1 | 1/1 | 0/1 | 0/1 | 0/1 | [19] |
| **Gross deletion** |  | |  |  |  |  |  |  |  |  |
| del3384bp | 1 | | F | 1/1 | 0/1 | 0/1 | 0/1 | 1/1 | 0/1 | [22] |
| del6Mb | 1 | | M | 1/1 | 0/1 | 0/1 | 0/1 | 0/1 | 0/1 | [7] |
| **Homozygous** |  | |  |  |  |  |  |  |  |  |
| c.3160C>T | 2 | | 2F | 1/1 | 0/2 | 1/2 | 2/2 | 0/2 | 0/2 | [23] |
| **Total** | 81 | | 37F/41M/3? | 19/81 | 23/81 | 52/81 | 11/81 | 12/81 | 10/81 |  |
| （%） |  | |  | 23% | 28% | 64% | 13% | 14% | 12% |  |

N: Number of the TBS patients; F: Female; M: Male; RF: Renal failure (Patients had received renal replacement therapy or creatinine >707μmol/L or eGFR≤15ml/min/1.73m^2^.); RFI: Renal function impaired (The patient's serum creatinine level is above the normal range but doesn't meet the diagnosis of renal failure); HPL: hypoplastic; PK: Polycystic kidney; VR: Vesicoureteral reflux; HSP: hypospadias.

**Reference**

1. Botzenhart, E., et al., *Townes-Brocks syndrome: twenty novel SALL1 mutations in sporadic and familial cases and refinement of the SALL1 hot spot region.* 2007. **28**(2): p. 204-5.

2. Kohlhase, J., et al., *Molecular analysis of SALL1 mutations in Townes-Brocks syndrome.* 1999. **64**(2): p. 435-45.

3. Surka, W., et al., *Unique family with Townes-Brocks syndrome, SALL1 mutation, and cardiac defects.* 2001. **102**(3): p. 250-7.

4. Salerno, A., J. Kohlhase, and B.J.P.n. Kaplan, *Townes-Brocks syndrome and renal dysplasia: a novel mutation in the SALL1 gene.* 2000. **14**(1): p. 25-8.

5. Faguer, S., et al., *Nephropathy in Townes-Brocks syndrome (SALL1 mutation): imaging and pathological findings in adulthood.* 2009. **24**(4): p. 1341-5.

6. Botzenhart, E., et al., *SALL1 mutation analysis in Townes-Brocks syndrome: twelve novel mutations and expansion of the phenotype.* 2005. **26**(3): p. 282.

7. Morisada, N., et al., *16q12 microdeletion syndrome in two Japanese boys.* 2014. **56**(5): p. e75-8.

8. Marlin, S., et al., *Townes-Brocks syndrome: detection of a SALL1 mutation hot spot and evidence for a position effect in one patient.* 1999. **14**(5): p. 377-86.

9. Choi, W., et al., *A family with Townes-Brocks syndrome with congenital hypothyroidism and a novel mutation of the SALL1 gene.* 2010. **53**(12): p. 1018-21.

10. Fang, J., et al., *SALL1Novel mutation in the gene in a four-generation Chinese family with uraemia: A case report.* 2022. **10**(20): p. 7068-7075.

11. Beaudoux, O., et al., *Adult diagnosis of Townes-Brocks syndrome with renal failure: Two related cases and review of literature.* 2021. **185**(3): p. 937-944.

12. Liberalesso, P., et al., *Phenotypic and genotypic aspects of Townes-Brock syndrome: case report of patient in southern Brazil with a new SALL1 hotspot region nonsense mutation.* 2017. **18**(1): p. 125.

13. Barry, J. and M.J.O.g. Reddy, *The association of an epibulbar dermoid and Duane syndrome in a patient with a SALL1 mutation (Townes-Brocks Syndrome).* 2008. **29**(4): p. 177-80.

14. van Bever, Y., et al., *Obstructive apneas and severe dysphagia in a girl with Townes-Brocks syndrome and atypical feet involvement.* 2009. **52**(6): p. 426-9.

15. Keegan, C., et al., *Townes-Brocks syndrome versus expanded spectrum hemifacial microsomia: review of eight patients and further evidence of a "hot spot" for mutation in the SALL1 gene.* 2001. **3**(4): p. 310-3.

16. Lin, F., et al., *SALL1Delayed diagnosis of Townes-Brocks syndrome with multicystic kidneys and renal failure caused by a novel nonsense mutation: A case report.* 2016. **11**(4): p. 1249-1252.

17. Albrecht, B., M. Liebers, and J.J.A.j.o.m.g.P.A. Kohlhase, *Atypical phenotype and intrafamilial variability associated with a novel SALL1 mutation.* 2004(1): p. 102-4.

18. van den Akker, P., et al., *Somatic mosaicism for the SALL1 mutation p.Ser371X in full-blown Townes-Brocks syndrome with Duane anomaly.* 2009(4): p. 812-5.

19. Blanck, C., et al., *Three novel SALL1 mutations extend the mutational spectrum in Townes-Brocks syndrome.* 2000. **37**(4): p. 303-7.

20. Reardon, W., et al., *Kidney failure in Townes-Brocks syndrome: an under recognized phenomenon?* 2007(21): p. 2588-91.

21. Walter, K., et al., *Mosaic trisomy 8 and Townes-Brocks syndrome due to a novel SALL1 mutation in the same patient.* 2006. **140**(6): p. 649-51.

22. Borozdin, W., et al., *Detection of heterozygous SALL1 deletions by quantitative real time PCR proves the contribution of a SALL1 dosage effect in the pathogenesis of Townes-Brocks syndrome.* 2006. **27**(2): p. 211-2.

23. Vodopiutz, J., et al., *Homozygous SALL1 mutation causes a novel multiple congenital anomaly-mental retardation syndrome.* 2013. **162**(3): p. 612-7.
